# Giant Uterine Leiomyoma With Concomitant Occult Ovarian Carcinoma: A Case Report

**DOI:** 10.7759/cureus.108829

**Published:** 2026-05-14

**Authors:** Stefanos Flindris, Effrosyni Styliara, Georgia Galaziou, Panagiotis Tsirkas, Konstantinos Dinas, Minas Paschopoulos, Iordanis Navrozoglou

**Affiliations:** 1 Department of Obstetrics and Gynecology, University Hospital of Ioannina, Ioannina, GRC; 2 Derpatment of Radiology, University Hospital of Ioannina, Ioannina, GRC; 3 Department of Obstetrics and Gynecology, General Hospital of Ioannina G. Hatzikosta, Ioannina, GRC; 4 2nd Department of Obstetrics and Gynecology, Gynecologic Oncology Unit, Ippokrateio General Hospital of Thessaloniki, Aristotle University of Thessaloniki, Thessaloniki, GRC

**Keywords:** clear cell, giant myoma, hysterectomy, ovarian cancer, uterine

## Abstract

Uterine leiomyomas are common benign tumors, whereas “giant” leiomyomas occupying most of the abdominal cavity are now uncommon and can pose diagnostic and operative challenges. We present the case of a 45-year-old woman with progressive abdominal distension and long-standing menorrhagia. Computed tomography demonstrated a massive uterine fundal mass consistent with a giant subserosal leiomyoma and a separate right adnexal cystic lesion. The patient underwent elective midline laparotomy and total abdominal hysterectomy with bilateral salpingo-oophorectomy. The ovarian cyst was removed intact without capsular rupture, and peritoneal washings were obtained. Estimated blood loss was 80 mL. Postoperative recovery followed an enhanced recovery after surgery (ERAS) pathway, enabling discharge on postoperative day four. Final histopathology confirmed a giant subserosal leiomyoma measuring 44 × 38 × 30 cm and revealed an occult clear cell carcinoma confined to the ovarian cyst (FIGO stage IA), with negative peritoneal cytology. Completion staging surgery and adjuvant-treatment discussion were recommended at a multidisciplinary tumor board; however, the patient declined further extensive surgery and was referred to medical oncology for adjuvant management and close surveillance. This case highlights the complexity of removal in giant uterine tumors, careful handling of adnexal cysts to avoid intraoperative rupture, and the feasibility of ERAS-based recovery.

## Introduction

Leiomyomas, or fibroids, are the most common benign pelvic tumors in women and arise monoclonally from the smooth muscle cells of the uterine myometrium [1]. These tumors occur in a substantial proportion of women over the age of 35 years, with increased prevalence during the reproductive years due to hormone-driven growth. Extremely large myomas may lead to serious complications, including respiratory compromise due to diaphragmatic elevation and compression of intrathoracic structures [1,2]. Although the diagnosis of a giant myoma can be challenging, with several possible differential diagnoses including ovarian neoplasms and other abdominopelvic masses, the majority of uterine myomas are still confidently recognized in routine clinical and preclinical practice [1,2].

Uterine leiomyomas may affect up to 70% of women by the end of reproductive life, depending on age, ethnicity, and diagnostic methodology [3]. While most fibroids remain small and asymptomatic, a considerable proportion of women develop symptoms such as abnormal uterine bleeding, pelvic pressure, pain, or infertility that ultimately require treatment [3]. Contemporary management encompasses a broad spectrum of medical, interventional, and surgical options, which are tailored to the patient’s symptom burden, tumor characteristics, comorbidities, and reproductive wishes [3,4].

In contrast, “giant” uterine leiomyomas are exceptionally rare in modern practice and are typically defined as tumors weighing ≥11.4 kg or exhibiting very large dimensions [4]. Fewer than 100 such cases have been reported worldwide, often in the context of delayed gynecologic assessment, limited access to care, or long-standing neglect of progressive abdominal enlargement [1,3,4]. As these tumors grow to massive dimensions, they can occupy the entire abdominopelvic cavity, displace or compress adjacent organs, and cause potentially life-threatening complications such as bowel obstruction, venous stasis, or respiratory compromise [3-5]. Preoperative imaging may not reliably distinguish giant benign leiomyomas from uterine sarcomas or large adnexal masses, and serum tumor markers such as CA-125 lack specificity in this setting [5].

Surgery remains the cornerstone of treatment for giant fibroids, with most published reports advocating open midline laparotomy and intact specimen removal, usually total abdominal hysterectomy with or without bilateral salpingo-oophorectomy to minimize tumor rupture and potential dissemination of an unsuspected malignancy [6]. Concurrently, the implementation of enhanced recovery after surgery (ERAS) protocols in gynecologic oncology and major benign gynecologic surgery has been associated with reduced postoperative length of stay, fewer complications, decreased opioid use, and lower healthcare costs without compromising safety [6].

We report the case of a 45-year-old woman with a giant subserosal uterine leiomyoma measuring 44 × 38 × 30 cm, successfully managed with open total abdominal hysterectomy and bilateral salpingo-oophorectomy within an ERAS pathway, highlighting the importance of a specialized gynecological oncology surgical team, meticulous preoperative planning, intact tumor removal, and structured perioperative optimization in the management of very large benign uterine tumors.

## Case presentation

A 45-year-old multiparous woman was referred to our gynecology department because of progressive abdominal distension and a palpable mass. Over the previous two years, she had noted progressively prolonged menstrual bleeding, with menses lasting up to 14 days but remaining roughly 28 days apart. She reported a sensation of abdominal “fullness” and mild urinary pressure, but denied acute abdominal pain, weight loss, changes in bowel habits, or constitutional symptoms. Her medical history was significant for arterial hypertension and type 2 diabetes mellitus, both treated with oral medication. She had a history of one cesarean section and an appendectomy. There was no known family history of gynecologic or breast malignancy. She was a light smoker (approximately six cigarettes per day) and had no prior blood transfusions.

**Figure 1:**
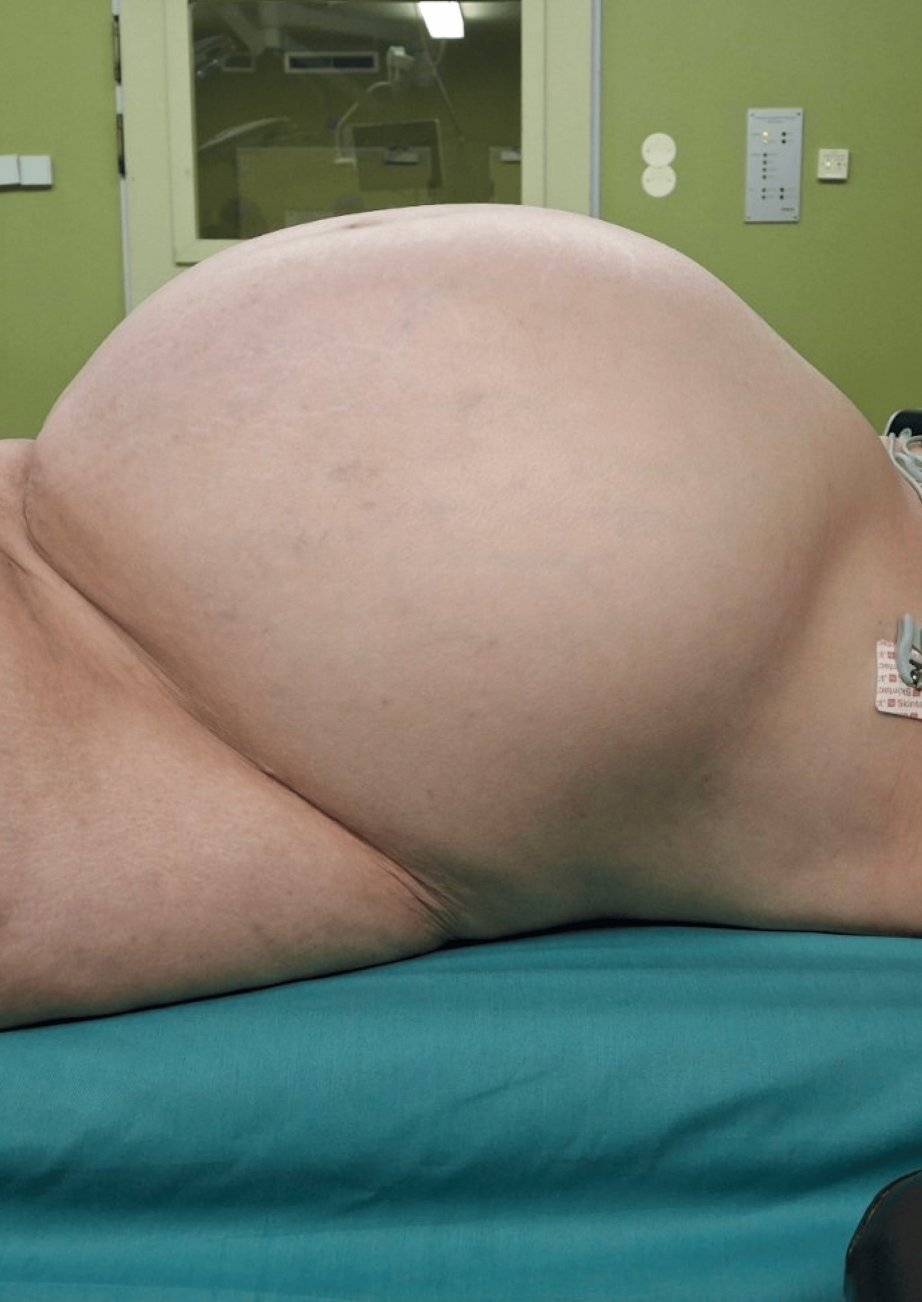
A lateral (profile) view illustrates the full extent of the uterine mass.

On physical examination, the patient was hemodynamically stable. The abdomen was markedly distended, and a large, firm, mobile, non-tender mass occupied almost the entire abdominal cavity, extending from the pelvis to the epigastrium (Figure 1). There were no signs of peritoneal irritation. On bimanual pelvic examination, the uterus felt markedly enlarged, while the cervix appeared macroscopically normal. No obvious adnexal tenderness or nodularity was detected. Baseline laboratory tests, including complete blood count, coagulation profile, renal function, and liver enzymes, were within normal limits. Serum β-human chorionic gonadotropin (β-hCG) was <0.5 mIU/mL. Tumor markers showed a mildly elevated CA-125 (39 U/mL), with normal CA 19-9, carcinoembryonic antigen (CEA), and CA 15-3. Preoperative blood tests and postoperative day (POD) 1 values are summarized in Table 1.

**Table 1:** Preoperative (POD0), postoperative day 1 (POD1), and discharge (POD6) laboratory values. CEA: Carcinoembryonic antigen

Parameter	Unit	Preoperative (POD0)	POD1 Value	POD6 (Discharge)	Reference Range (Adult Female)
White blood cells	×10³/µL	6.8	11.47	8.15	4.0–10.0
Neutrophils	%	62.0	77.4	60.5	40–75
Hemoglobin	g/dL	13.1	12.4	12.9	12.0–16.0
Hematocrit	%	40.3	37.8	36.2	36–46
Platelets	×10³/µL	225	221	328	150–400
Prothrombin time (PT)	seconds	12.8	12.5	12.6	11–15
INR	–	0.96	0.96	0.98	0.8–1.2
Activated partial thromboplastin time (aPTT)	seconds	28	29	27	25–35
Fibrinogen	mg/dL	352	370	340	200–400
Glucose (serum)	mg/dL	97	94	143	70–100 (fasting)
Urea	mg/dL	32	34	38	15–45
Creatinine	mg/dL	0.72	0.71	0.70	0.5–1.1
Sodium	mmol/L	138	137	138	135–145
Potassium	mmol/L	4.38	4.10	4.54	3.5–5.1
Aspartate aminotransferase (AST)	U/L	12	13	13	0–35
Alanine aminotransferase (ALT)	U/L	11	11	12	0–35
Serum cholinesterase (CHE)	kU/L (lab-specific)	7	–	7.1	Lab-specific
β-hCG	mIU/mL	<0.5	–	–	<5 (non-pregnant)
CA-125	U/mL	39	–	–	0–35
CA 19-9	U/mL	8	–	–	0–37
CEA	ng/mL	0.9	–	–	<5
CA 15-3	U/mL	12	–	–	0–25

**Figure 2:**
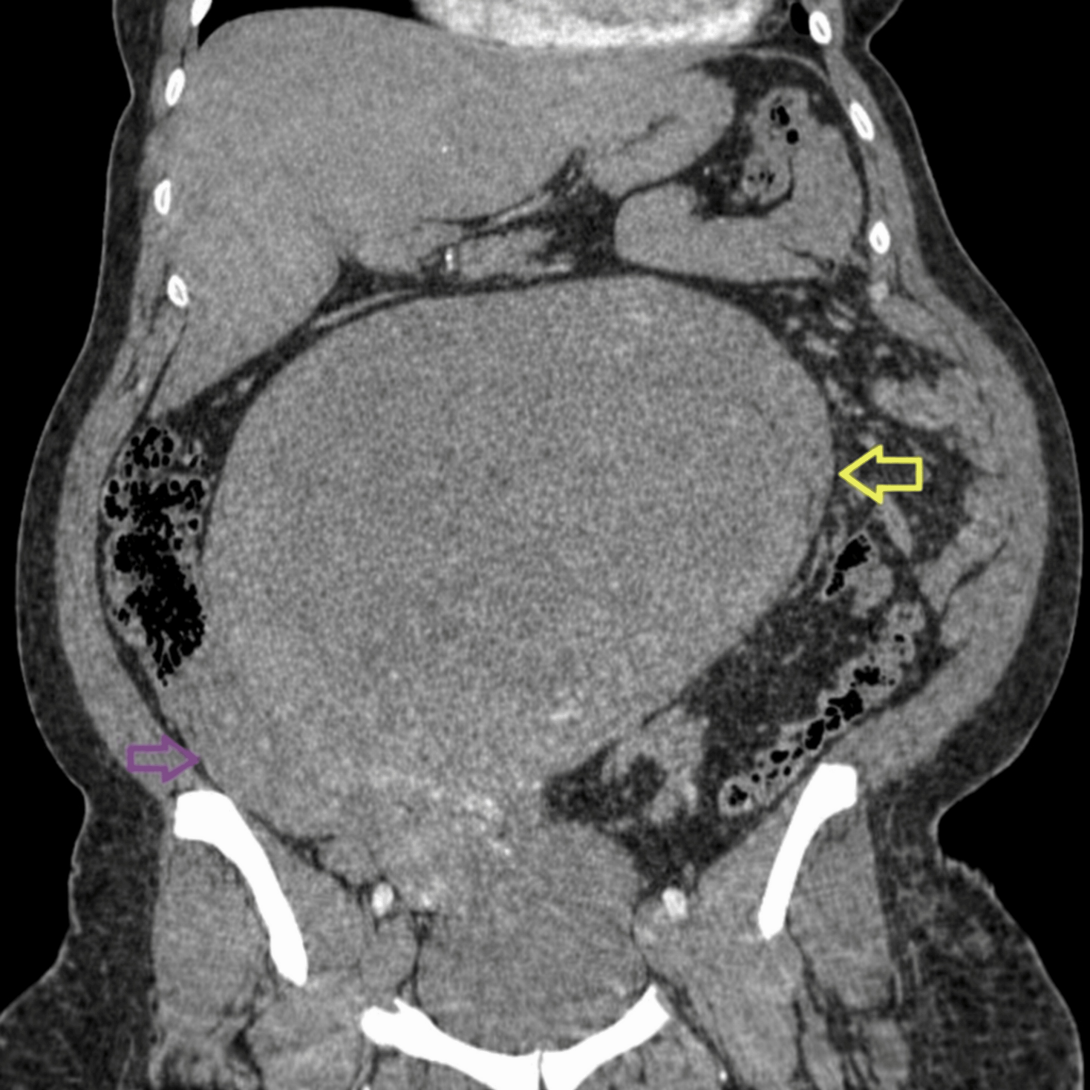
Contrast-enhanced coronal CT of the abdomen demonstrates a large uterine leiomyoma (yellow arrow) and an adjacent cystic lesion of the right ovary (purple arrow).

**Figure 3:**
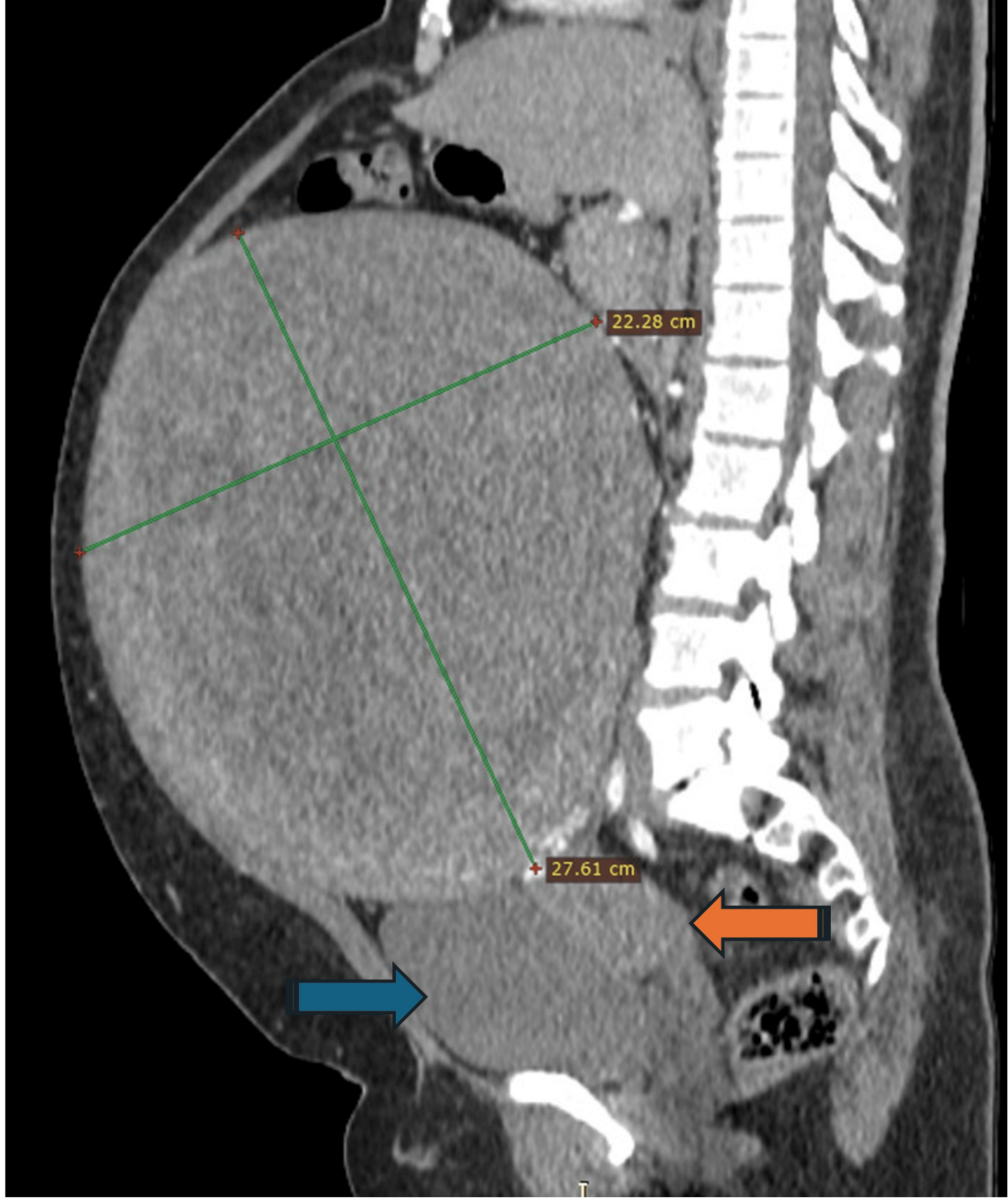
Contrast-enhanced sagittal CT demonstrates a giant uterine subserosal leiomyoma arising from the uterine fundus, measuring 27.61 × 22.28 cm. Blue arrow: urinary bladder and orange arrow: uterus.

**Figure 4:**
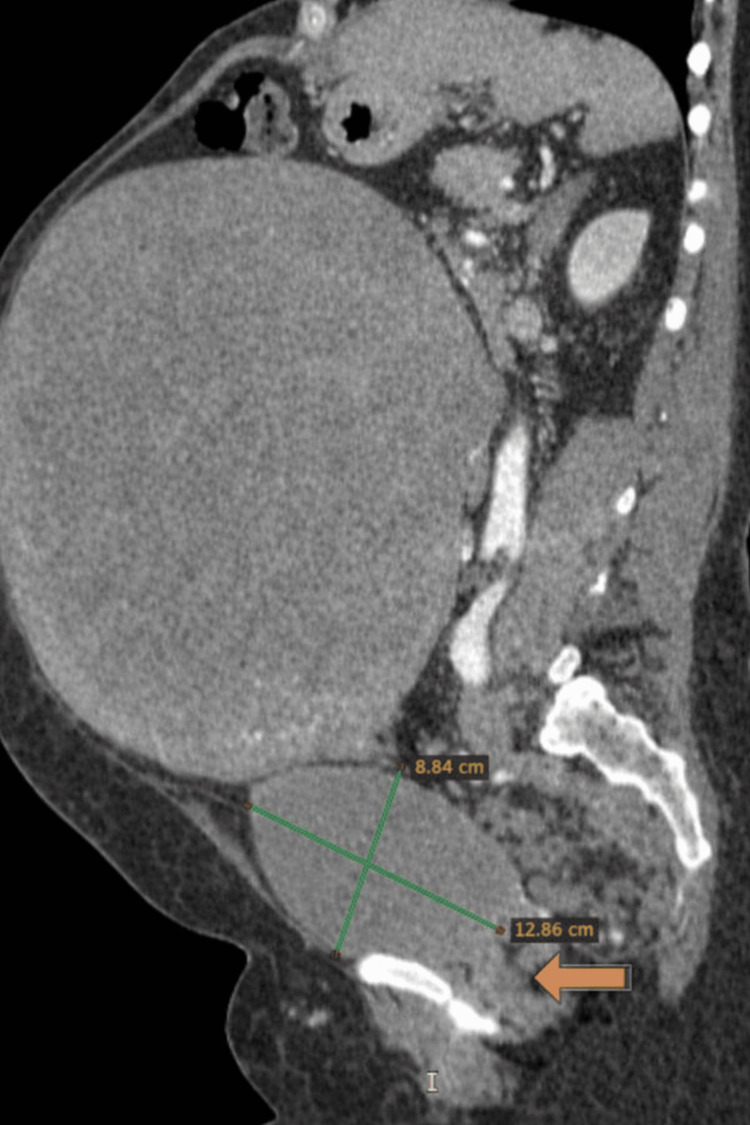
Right parasagittal contrast-enhanced CT image demonstrates caliper measurements of a cystic mass measuring 12.86 × 8.84 cm arising from the right adnexa.

Contrast-enhanced computed tomography (CT) of the abdomen and pelvis revealed a huge, well-circumscribed mass 40 x 38 x 29 cm occupying most of the peritoneal cavity, displacing but not invading adjacent organs and appearing to arise from the uterine fundus (Figures 2, 3). The radiologic impression was that of a giant subserosal leiomyoma. A cystic lesion in the right adnexa, compatible with a hemorrhagic ovarian cyst, was also identified, 12 x 9 x 7cm (Figure 4). No ascites or suspicious para-aortic or pelvic lymphadenopathy were noted. Given the size of the mass, the patient’s comorbidities, and the expected magnitude of surgery, the case was discussed in a multidisciplinary setting. Anesthesiology assessment documented an increased perioperative risk and recommended that an intensive care unit (ICU) bed be available postoperatively if needed. After informed consent, elective surgery via laparotomy was planned.

**Figure 5:**
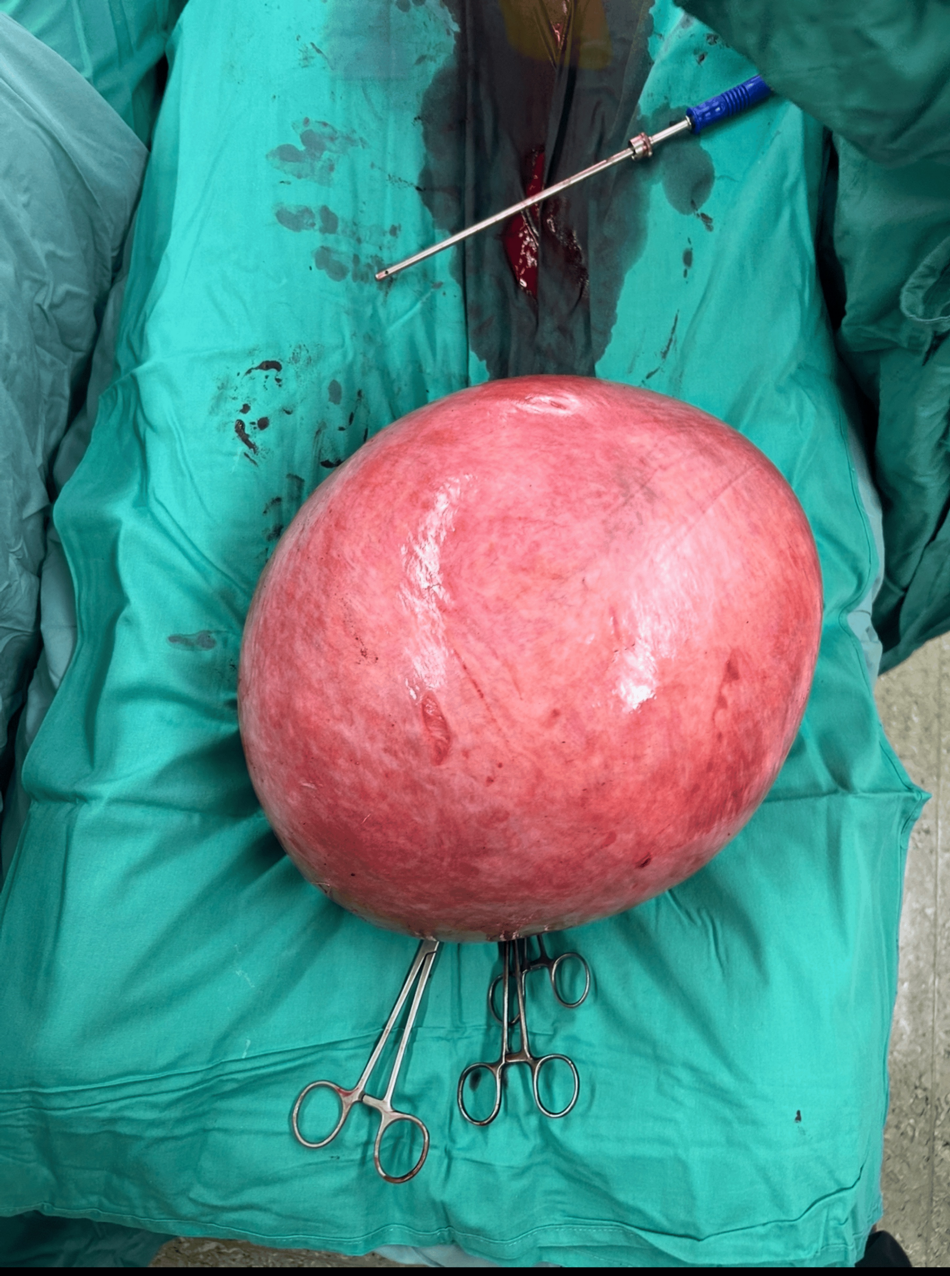
The surgical specimen of the resected uterine mass.

Under general anesthesia, a midline vertical incision from the pubic symphysis to above the umbilicus was performed to ensure adequate exposure. On entering the peritoneal cavity, peritoneal washing was obtained for cytologic examination. A massive uterine tumor was visualized, filling almost the entire abdominal cavity and arising from the uterine fundus. The right ovary contained a large cystic lesion, corresponding to the CT finding. No macroscopic evidence of peritoneal implants or extrauterine disease was observed. The mass was carefully mobilized and exteriorized to facilitate safe handling. A total abdominal hysterectomy with bilateral salpingo-oophorectomy was performed by a gynecologic oncologist. The uterine vessels and supporting ligaments were ligated in a stepwise fashion, the uterus was transected at the isthmus, and the cervix and the right adnexa with the cystic lesion were removed separately. Hemostasis was meticulous, peritoneal irrigation was carried out, and no drains were placed. Estimated blood loss was approximately 80 mL, and no intraoperative complications or transfusions were required. The weight of the mass was calculated to be 12, 195 g. The surgical specimen of the excised uterine mass is depicted in Figure 5.

Postoperative recovery followed an ERAS protocol. The patient received standard thromboprophylaxis and antibiotic prophylaxis, was mobilized on POD 1, and tolerated early oral intake. Laboratory testing on POD1 showed mild leukocytosis with neutrophilia, stable hemoglobin, and preserved renal and hepatic function. Pain was adequately controlled with multimodal analgesia. She had no postoperative fever, ileus, or wound complications and was discharged home in good condition on POD6 with instructions for continued thromboprophylaxis and use of an abdominal binder. Quadriceps strength was assessed using a pull-type handheld dynamometer preoperatively at six weeks and one week. It was also assessed postoperatively at four days, one month, and three months. This modification clarifies the method of muscle strength assessment

Gross pathological examination of the specimen revealed a markedly enlarged uterus with an attached subserosal mass measuring 44 × 38 × 30 cm, confirming a giant uterine leiomyoma. The right ovarian cystic lesion, which had been removed intact without intraoperative rupture, was found to harbor an occult clear cell carcinoma confined to the cyst (FIGO stage IA). Peritoneal cytology was negative for malignant cells. After discussion at a multidisciplinary gynecologic oncology meeting, completion staging surgery (including omentectomy, systematic pelvic and para-aortic lymphadenectomy as well as peritoneal biopsies) was recommended; however, the patient declined further extensive surgical staging and opted instead for referral to a medical oncologist to proceed with adjuvant chemotherapy treatment and close oncologic follow-up.

## Discussion

Uterine leiomyomas are the most common benign pelvic tumors in women, with a lifetime prevalence approaching 70% in some populations [3]. Most fibroids remain small and asymptomatic; only a minority cause significant bleeding, bulk symptoms, or hematologic morbidity [7]. By contrast, “giant” leiomyomas, usually defined as fibroids weighing ≥11.3-11.4 kg or measuring more than 17 cm, are rare in contemporary practice [4,8]. The leiomyoma in our patient, measuring 44 × 38 × 30 cm, clearly belongs to this extreme category in terms of abdominal occupation, even if the exact weight is below the historical record tumors exceeding 45-60 kg [4,9].

Clinical presentation of giant leiomyomas varies widely, from incidental findings to severe anemia and organ compression [10]. Some patients remain asymptomatic for years despite tumors of 10-16 kg, whereas others present with massive uterine enlargement, respiratory compromise, or transfusion-dependent menorrhagia [7,10]. Our patient had long-standing menorrhagia and progressive abdominal distension but preserved hemoglobin and only mild pressure symptoms, illustrating how the compliance of the peritoneal cavity can mask very large tumors and delay diagnosis.

Preoperative characterization of large uterine masses is difficult. Ultrasound is the first-line imaging modality for suspected leiomyomas, but degenerative changes (hyaline, cystic, red, or calcific) can obscure the typical myoma appearance and mimic ovarian or other abdominopelvic tumors [10]. Some studies of giant fibroids repeatedly show that CT or MRI may fail to clearly delineate tumor origin or to exclude sarcoma, especially when the mass fills the abdomen and distorts normal anatomy [10,11]. Furthermore, benign leiomyomas may be associated with mild CA-125 elevation, limiting the specificity of tumor markers [5,11]. In our patient, contrast-enhanced CT correctly suggested a giant subserosal fundal mass and a separate hemorrhagic ovarian cyst, but malignancy could not be definitively ruled out preoperatively.

In recent years, several algorithms have been proposed to improve preoperative risk stratification for uterine sarcoma. The Uterine mass Magna Graecia (U.M.G.) risk score combines clinical variables and serum lactate dehydrogenase isoenzymes into a numerical index; values ≥29 are associated with a substantially increased probability of uterine sarcoma [12]. Initial and subsequent validation studies reported high specificity and negative predictive value for excluding sarcoma in women with uterine masses, suggesting that the U.M.G. score could guide the choice between minimally invasive surgery with morcellation versus open, non-morcellated removal [12,13]. In parallel, the Basel Sarcoma Score (BSS) was developed as an ultrasound-based tool using six sonographic criteria (rapid growth, high blood flow, atypical growth, irregular endometrial lining, central necrosis, and solitary oval lesion) [14]. In a prospective cohort of 545 women with myoma-like masses, a BSS >1 achieved sensitivity of 93.8%, specificity of 97.9%, and a negative predictive value of 99.8% for sarcomatous lesions [14]. Although neither score can absolutely exclude malignancy, current ESGO/EURACAN uterine sarcoma guidelines acknowledge the potential role of such validated indices as part of a structured preoperative assessment [15].

In our patient, the decision for open midline laparotomy and intact specimen removal was based on tumor size, age, completed childbearing, and the impossibility of confidently excluding malignancy. This approach is consistent with published series and case reports of giant leiomyomas, which overwhelmingly favor midline laparotomy with total abdominal hysterectomy (with or without bilateral salpingo-oophorectomy) to ensure adequate exposure and avoid morcellation. Given the catastrophic consequences of inadvertent morcellation of an occult sarcoma and the still-limited performance of all available imaging and biochemical tests, major societies continue to recommend extreme caution and avoidance of power morcellation when malignancy cannot be excluded [15].

Despite the technical demands of giant fibroid surgery, our patient’s perioperative course was favorable, in which the estimated blood loss was only 80 mL, no transfusion or bowel/urinary tract injury occurred, and histology confirmed a benign leiomyoma without atypia, coagulative necrosis, or elevated mitotic activity. This compares favorably with other reports in which large blood loss, transfusion, and even bowel resection are not uncommon [2,9,10]. An additional strength of our case is the application of an ERAS pathway incorporating early mobilization, multimodal analgesia, rapid diet advancement, and standardized thromboprophylaxis, which allowed safe discharge on POD 4 [16]. Multiple observational and randomized studies in gynecologic surgery have shown that ERAS programs reduce length of stay and complications without increasing readmission or compromising safety.

Uterine artery embolization (UAE) has been explored as a uterus-sparing option for very large (“giant”) fibroid burdens, but the evidence base is more limited than for typical-size leiomyomas. In a systematic review and meta-analysis of women with giant fibroids (commonly defined as dominant fibroid ≥10 cm and/or uterine volume ≥700 cm³), UAE achieved meaningful reductions in fibroid/uterine volume and was considered safe and effective, albeit with significantly higher major-complication and reintervention rates compared with non-giant fibroids, highlighting the need for careful patient selection and counseling [17].

An additional critical aspect of this case was the incidental diagnosis of an occult clear cell carcinoma confined to the right ovarian cyst (FIGO IA). Intact removal of the cyst without capsular rupture and negative peritoneal cytology were essential to avoid immediate upstaging to FIGO IC1, which would carry a higher risk of relapse and clearly stronger indications for adjuvant chemotherapy according to ESGO-ESMO consensus recommendations [18]. Current guidelines state that apparent early-stage epithelial ovarian cancer, especially clear cell histology, should undergo full surgical staging (including systematic lymphadenectomy, omentectomy, peritoneal biopsies, and systematic inspection of the peritoneal cavity), and they consider omission of adjuvant chemotherapy only in completely staged stage IA/IB/IC1 disease [18]. Given that our patient had not undergone formal staging beyond hysterectomy, bilateral salpingo-oophorectomy, washings, and intact cystectomy, the multidisciplinary tumor board recommended completion staging surgery and discussion of platinum-based adjuvant chemotherapy in line with these ESGO-ESMO principles, while acknowledging that the absolute benefit of chemotherapy in fully staged FIGO IA clear cell carcinoma remains uncertain [19]. The patient ultimately declined further extensive surgery and elected referral to a medical oncologist for systemic-therapy counselling and close surveillance, underlining the need to balance guideline-concordant care with informed patient preference when an unexpected early ovarian carcinoma is discovered after surgery for presumed benign disease.

Very large uterine masses warrant a deliberately cautious surgical strategy because uterine leiomyosarcoma outcomes are largely determined by stage and grade and retrospective data suggest limited survival benefit from adjuvant modalities, which makes careful primary surgery and avoidance of tumor disruption particularly important [15,20]. Although minimally invasive hysterectomy can be feasible for benign uteri weighing more than 1 kg in expert centers and is associated with lower blood loss and shorter hospitalization, this approach is not inevitable in giant tumors, where exposure, safe specimen retrieval, and the need to avoid morcellation may mandate laparotomy. In our case, the extreme uterine size together with the unexpected occult adnexal carcinoma on final pathology reinforces the value of planning for intact en bloc removal and meticulous handling of adnexal cysts without rupture, so that surgical upstaging is avoided and subsequent oncologic management remains optimized [20].

## Conclusions

In conclusion, this case underscores that deficiencies in routine gynecologic surveillance may allow uterine leiomyomas to attain extreme dimensions before clinical intervention. The patient had not undergone gynecologic evaluation or cervical screening for five years, despite progressive menorrhagia and increasing abdominal distension. Comparable delays, attributable to reduced access to care, under-recognition of symptom significance, or patient-related factors, have been described in reports of giant fibroids. Early evaluation of abnormal uterine bleeding and abdominal enlargement, combined with structured preoperative risk stratification using instruments such as the U.M.G. score or the BSS when indicated, together with adjunctive imaging modalities such as pelvic MRI, may facilitate earlier diagnosis, improve identification of lesions with features suspicious for uterine sarcoma, and support optimal selection of the surgical approach while maintaining oncologic safety.
